# Pregabalin as a co-analgesic in patients undergoing laparoscopic sleeve gastrectomy: a double-blind, randomized, prospective clinical trial

**DOI:** 10.1038/s41598-026-47779-x

**Published:** 2026-04-06

**Authors:** Piotr Mieszczański, Marcin Jurczak, Marek Janiak, Grzegorz Górniewski, Paweł Ziemiański, Radosław Cylke, Paweł Skrzypek, Izabella Godlewska, Janusz Trzebicki

**Affiliations:** 1 1st Department of Anesthesiology and Intensive Care, Medical University of Warsaw, ul. Lindleya 4, 02-005 Warsaw, Poland; 2 Department of General Surgery and Transplantology, Medical University of Warsaw, ul. Nowogrodzka 59, 02-006 Warsaw, Poland

**Keywords:** Diseases, Health care, Medical research

## Abstract

Obese patients undergoing bariatric surgery are particularly susceptible to increased postoperative pain and opioid-related adverse effects. Enhancing patient safety and comfort may include the reduction of opioid requirements by the use of co-analgesics such as pregabalin. The aim of our study was to evaluate its safety and efficacy in patients undergoing laparoscopic sleeve gastrectomy (LSG). This double-blind, randomized controlled trial involved 90 patients who were assigned to receive either pre-operative pregabalin 150 mg or a placebo. The primary outcome was total oxycodone consumption within the first 24 h. We also assessed pain scores, incidence of nausea and vomiting, pregabalin-related side effects, hemodynamic parameters, and recovery at predefined time points. The pregabalin group had a similar total oxycodone consumption compared to the placebo. Concerning pain assessment, the study group had a lower score at the 6th hour, 2.16 vs. 2.64 (*p* = 0.047). No differences were found in the other outcomes. Pre-emptive 150 mg pregabalin before LSG was well tolerated, yet did not significantly reduce total oxycodone consumption. Moreover, the observed differences between groups at specific time points do not support the routine use of pregabalin until further research demonstrates clinically significant benefits.

## Introduction

Bariatric surgery remains one of the most effective interventions for achieving substantial and sustained weight loss in patients with severe obesity. However, procedures such as laparoscopic sleeve gastrectomy (LSG) may lead to moderate-to-severe postoperative pain requiring increased opioid use^1^. Opioids may be effective at reducing intense postoperative pain, but cause undesirable side effects such as respiratory depression or ileus and impact patient safety and comfort of the susceptible obese patient^2^. The accepted standard of care utilizes multimodal analgesia to target various points of the pain pathway, improve recovery, and reduce the need for opioids by employing co-analgesics as well as regional anesthesia techniques^3^.

Pregabalin is a γ-aminobutyric acid (GABA) analogue that binds to α2δ subunits of the voltage-gated calcium channels and diminishes the excitability of dorsal horn sensory neurons subsequent to tissue damage. It is regarded as a potential co-analgesic with opioid-sparing, anti-hyperalgesic properties^4^ and may serve as an initial treatment for neuropathic pain^5^. Neural injury responses resulting from surgical dissection and coagulation constitute additional factors contributing to pain following laparoscopic bariatric surgery, which may be mitigated through the use of pregabalin.

The updated Procedure-Specific Postoperative Pain Management (PROSPECT) guidelines in LSG^6^ delineate promising results from two randomized controlled trials (RCT) in which gabapentin, a drug with a mechanism of action similar to pregabalin, was assessed for pain management following LSG^4,7^, showing the need for further research into the use of GABA agonists for the treatment of acute pain.

To address this knowledge gap, we conducted an RCT to test the hypothesis that pregabalin, in comparison with a placebo, would reduce the total postoperative opioid consumption and the severity of opioid-related side effects, while enhancing pain scores and recovery in patients undergoing LSG.

## Methods

### Trial design

This study was approved by the Bioethics Committee of the Medical University of Warsaw (KB/17/2023) and registered on 07.04.2023 in Clinical Trials (NCT05804591). The trial protocol was drafted in accordance with the Standard Protocol Items: Recommendations for Interventional Trials (SPIRIT) and published in an international peer-reviewed journal^8^. The study remains compliant with the principles outlined in the Declaration of Helsinki and adheres to the applicable Consolidated Standards of Reporting Trials (CONSORT) guidelines.

### Eligibility criteria

Adult patients scheduled for LSG with a BMI > 40 or > 35 with comorbidities, aged 18 to 65 years, were considered for the trial. Exclusion criteria were: unwilling to participate, scheduled for revision surgery, allergy to pregabalin, end-stage organ failure, or inability to use a patient-controlled analgesia (PCA) pump. Written informed consent was obtained from all participants of the trial by a study investigator.

### Conduct of the study

The study is a prospective, double-blind RCT conducted at a tertiary care teaching hospital between April 2023 and April 2025. Randomization was performed in a block with a parallel 1:1 allocation using the platform http://www.randomization.com (Dallal GE). The intervention consisted of administering an oral capsule of pregabalin 150 mg one to two hours prior to surgery, while the control group received an identically looking placebo capsule.

One hour prior to the surgical procedure, all patients received intravenously: 1 g of paracetamol, 2.5 g of metamizole, and 8 mg of dexamethasone. Anesthesia was induced with propofol at 2–2.5 mg/kg, and maintenance was provided with desflurane. Remifentanil, administered via Target Controlled Infusion (TCI) applying Minto Mode, was dosed to maintain hemodynamic stability. The depth of anesthesia was monitored through the Bispectral Index (BIS), with a target range of 40–60. Neuromuscular blockade was initiated and maintained with rocuronium under Train-of-Four (TOF) guidance and was reversed at the end of the procedure with intravenous sugammadex.

Local infiltration of the trocar insertion sites with 0.25% bupivacaine (40 ml total) was performed by the surgeon intraoperatively. After wound closure, remifentanil was discontinued, and oxycodone was administered at a dose of 0.1 mg/kg of ideal body weight (IBW) i.v. in all cases.

Following extubation, patients were transferred to the postanesthesia care unit (PACU), where postoperative care was provided in accordance with a standardized ERAS protocol. No drains or nasogastric tubes were implemented. Simple analgesics, paracetamol and metamizole were administered i.v. All patients had oxycodone on-demand analgesia (PCA, bolus 2 mg, lockout 10 min). Oxygen therapy was initiated upon PACU arrival and continued as a standard for the first two hours. A clear liquid regimen was initiated two hours after surgery. After an hour of observation in PACU, all patients were initially placed in a sitting position and then mobilized. The PACU discharge criteria included independence from oxygen therapy, pain less than 4 on the NRS scale, hemodynamic stability, mobilization, and absence of oversedation.

### Outcome measures

The primary outcome measure was the total oxycodone consumption in 24 h after surgery. Secondary, postoperative outcome measures were as follows: the pain scores on the NRS scale at 1st, 6th, 12th, and 24th hour after surgery, the sedation on the Ramsay scale^9^, the PONV impact scale^10^, the incidence of desaturations (SpO2 < 94%), the episodes of blurred vision at 1st, 6th, 12th and 24th hour after surgery and patient’s comfort assessed in QoR-40 questionnaire^11^ at discharge. Intraoperative outcome measures: maximal and minimal heart rate (HR), maximal and minimal blood pressure measurements: systolic (SBP), diastolic (DBP) and mean arterial blood pressures (MAP), time when MAP remained < 65 mmHg and > 90 mmHg, episodes of HR < 50 bpm and > 90, total fluid volume administered, in addition the total ephedrine dose administered during the surgery and in the postoperative period.

### Sample size calculation

The sample size was calculated for the primary outcome of the study, with the assumption that a difference exceeding 30% in total oxycodone consumption between the groups (δ) would be deemed a minimal clinically important difference (MCID)^12^. Utilizing a sample size calculation method suitable for two-tailed testing, as recommended^13^, a total of 76 participants would be adequate to identify a meaningful difference. To improve the robustness of the study and account for potential attrition, we have adopted a rounded sample size of 90 patients.

### Statistical analyses

The statistical analysis plan was integrated into the study protocol and published in a peer-reviewed journal before completion of our RCT^8^.

Descriptive statistics were reported for baseline characteristics and postoperative endpoints to provide an overview of group comparability and temporal trends. For demographics, anthropometric measures, intraoperative parameters, hemodynamic variables, and postoperative outcomes, categorical variables were summarized as counts and percentages with 95% CIs calculated using the Wilson score method. Continuous variables were presented as medians with 95% CI derived via the Wilcoxon method. For numeric clinical endpoints, including sedation score, nausea score, vomiting score, and PONV impact scale score, means with standard deviations were used due to their low-scale nature (mostly ordinal but analyzed as continuous) and to maintain consistency. Between-group comparisons for baseline data utilized Pearson’s chi-squared test for categorical variables and the Wilcoxon rank-sum test for continuous variables, employing a two-tailed significance threshold of *p* < 0.05. For extended between-group comparisons, a linear mixed-effects modeling approach was employed^13^. Missing data were assumed to be missing at random and handled by a linear mixed-effects model. All analyses were conducted utilizing the R statistical programming language (version 4.3.3; R Core Team, 2024) on the Windows 11 Pro 64-bit platform (build 26100).

## Results

### Study population

**Fig. 1:**
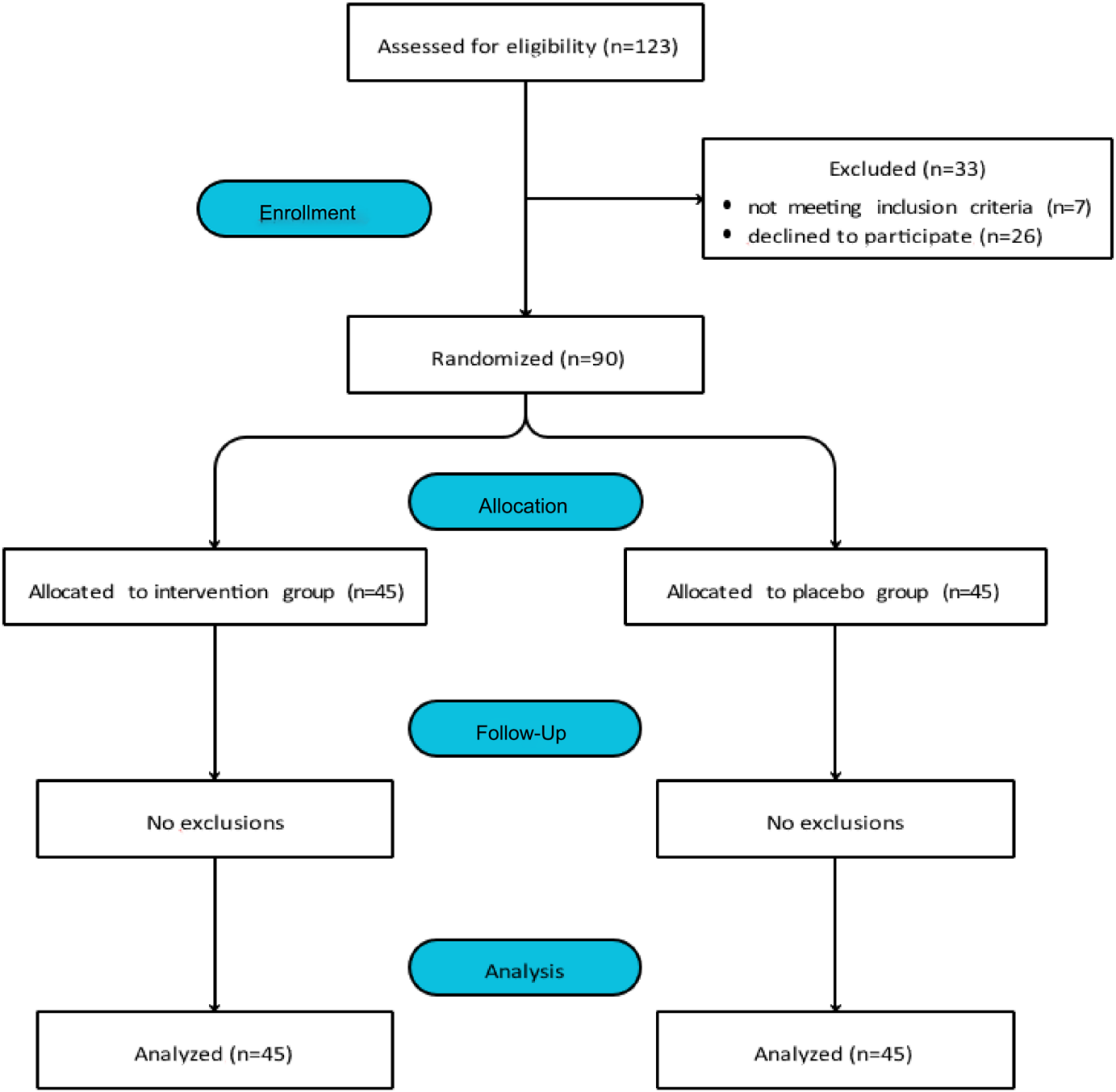
CONSORT flow diagram illustrating participant recruitment, randomization, and allocation.

Of the 123 patients assessed for eligibility, seven did not meet the inclusion criteria, and 26 declined to participate. The remaining 90 were randomized into two groups. All patients were analyzed as shown in Fig. 1.

Baseline characteristics demonstrated considerable comparability between both study arms (Table 1), with minor, negligible differences in anthropometric parameters: the pregabalin group had a higher median BMI of 43.0 kg/m² compared to 40.0 kg/m² (*p* = 0.015), and a greater median weight of 123.0 kg versus 113.0 kg (*p* = 0.043), as illustrated in Table 1. No significant differences were observed in anesthesia and surgery duration, and in both groups, no patients required urgent re-operation due to severe surgical complications during the assessed post-operative period.

**Table 1:** Baseline characteristics by treatment. Data expressed as numbers and percentages or medians, and 95% CI.

Characteristic	Pregabalin 150 mg (*N* = 45)	Placebo (*N* = 45)
Age (years)	44.0 (40.0–46.0)	42.0 (39.0–46.0)
Sex		
Female	30 -> 66.7% (51.0%, 80.0%)	30 -> 68.2% (52.0%-81.0%)
Male	15 -> 33.3% (20.0%-49.0%)	14 -> 31.8% (19.0%-48.0%)
Weight (kg)	123.0 (119.0-134.0)	113.0 (109.0-123.0)
Ideal Body Weight (kg¹)	65.0 (63.0–71.0)	63.0 (61.0–69.0)
Body Mass Index (kg/m²)	43.0 (42.0–45.0)	40.0 (39.0–42.0)
Anesthesia Duration (min)	100.0 (96.0-110.0)	98.0 (94.0-106.0)
Operation Duration (min)	75.0 (73.0–87.0)	75.0 (73.0–85.0)

### Primary outcome: oxycodone consumption

**Fig. 2:**
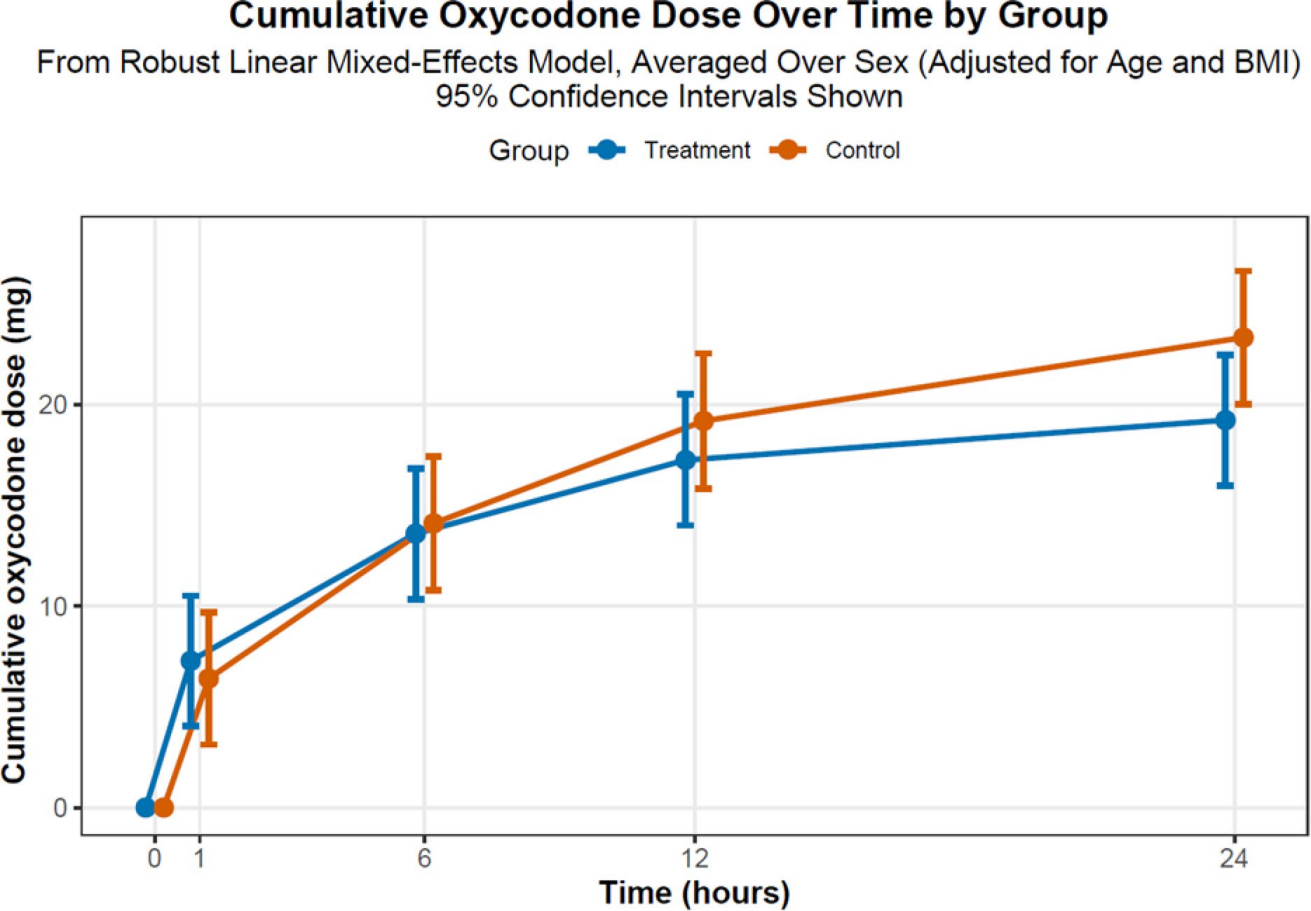
Estimated marginal means of cumulative oxycodone dose over 24 h by treatment (pregabalin 150 mg intake) and control (placebo) group in patients undergoing LSG.

There were no statistically significant differences between groups in total oxycodone consumption, with a median of 24.0 mg (Q1-Q3: 15.0-37.5) for pregabalin and 29.0 mg (Q1-Q3: 20.0–42.0) for placebo (*p* = 0.279). The linear mixed-effects model indicated comparable cumulative oxycodone doses (Fig. 2) at the predefined postoperative time points. In covariate analysis, advancing age correlated with lower oxycodone utilization (-0.34 mg per year beyond the mean of 43.50 years; *p* = 0.004), whereas neither sex nor body mass index exerted discernible effects (*p* > 0.05, linear mixed effects model). Nonetheless, pairwise group contrasts, tempered by Holm-Bonferroni corrections to mitigate multiplicity, failed to achieve statistical significance for differences in oxycodone dose between groups. Moreover, multivariable models were fitted with fixed effects including treatment group (pregabalin 150 mg intake vs. placebo), time points (1, 6, 12, and 24 h), their interaction, sex, age, and body mass index (BMI) and numeric covariates (age and BMI) were centered by subtracting the overall sample medians to enhance clinical interpretability. Observed BMI imbalance between groups did not influence the primary endpoints.

### Secondary outcomes

The NRS scores indicated a minor improvement in pain levels in the pregabalin group, with a score of 2.16 at the 6th hour compared to 2.64 (*p* = 0.047). There were no significant differences in other secondary parameters analyzed at predefined time points (see Table 2). Furthermore, there were no significant differences in intraoperative and postoperative secondary outcomes, except for the duration with MAP < 65 mmHg during surgery and the lowest observed MAP, both of which were more marked in the pregabalin group (see Table 3).

**Table 2:** Descriptive statistics for secondary clinical endpoints over time by treatment group in patients undergoing laparoscopic sleeve gastrectomy. Data are presented as mean (SD) for continuous variables or n (%) for categorical variables. NRS – numerical rating scale, PONV – postoperative nausea and vomiting.

Clinical parameter	Group	1 h	6 h	12 h	24 h
NRS score	Pregabalin 150 mg	4.41 (2.09)	2.64 (2.16)	2.05 (2.12)	2.07 (1.80)
	Placebo	5.36 (2.25)	3.53 (2.06)	2.81 (2.04)	2.11 (2.14)
Sedation score	Pregabalin 150 mg	2.44 (0.63)	2.15 (0.53)	2.18 (0.51)	2.00 (0.00)
	Placebo	2.41 (0.58)	2.14 (0.42)	2.10 (0.30)	1.93 (0.45)
Nausea score	Pregabalin 150 mg	0.82 (0.83)	0.67 (0.92)	0.50 (0.80)	0.41 (0.73)
	Placebo	1.16 (1.06)	0.86 (0.99)	0.57 (0.89)	0.44 (0.76)
Vomiting score	Pregabalin 150 mg	0.24 (0.57)	0.37 (0.87)	0.12 (0.33)	0.11 (0.44)
	Placebo	0.14 (0.35)	0.44 (0.88)	0.26 (0.70)	0.22 (0.67)
PONV impact scale score	Pregabalin 150 mg	1.07 (1.25)	1.05 (1.65)	0.62 (1.01)	0.52 (1.09)
	Placebo	1.27 (1.19)	1.30 (1.61)	0.81 (1.40)	0.67 (1.35)
Visual disturbances	Pregabalin 150 mg	5 (11.11%)	1 (2.33%)	1 (2.38%)	0 (0.00%)
	Placebo	1 (2.27%)	0 (0.00%)	0 (0.00%)	1 (2.27%)
Desaturation < 94%	Pregabalin 150 mg	7 (15.91%)	1 (2.38%)	1 (2.50%)	0 (0.00%)
	Placebo	4 (9.30%)	1 (2.50%)	1 (2.56%)	(0.00%)

**Table 3:** Descriptive statistics for secondary clinical endpoints. Data expressed as median and 95% CI. MAP – mean arterial pressure.

Clinical parameter	Pregabalin 150 mg	Placebo	*P*
*Intraoperative parameters*			
Time to extubation (min)	5.0 (4.5–6.5)	4.0 (CI: 3.5, 5.0)	0.077
Remifentanil dose (mcg/kg)²	4.2 (3.8–4.7)	4.9 (4.3–5.6)	0.122
Total fluid intake (mL)	750.0 (675.0-800.0)	750.0 (600.0-750.0)	0.193
Administered ephedrine (mg)	10.0 (12.0–20.0)	10.0 (12.0, 20.0)	0.249
Highest heart rate (bpm)	88.0 (86.0–94.0)	90.0 (84.0–93.0)	0.686
Highest systolic blood pressure (mmHg)	131.0 (127.0-135.0)	133.0 (129.0-140.0)	0.388
Highest diastolic blood pressure (mmHg)	82.0 (77.0–85.0)	84.0 (81.0–90.0)	0.174
Highest mean arterial pressure (mmHg)	99.0 (94.0-101.0)	97.7 (97.0-106.0)	0.199
Lowest heart rate (bpm)	63.0 (60.0–68.0)	65.0 (60.0–67.0)	0.654
Lowest systolic blood pressure (mmHg)	83.0 (80.0–88.0)	88.0 (85.0–93.0)	0.088
Lowest diastolic blood pressure (mmHg)	48.0 (47.0–52.0)	51.0 (49.0–54.0)	0.168
Lowest mean arterial pressure (mmHg)	60.0 (58.0–63.0)	63.0 (62.0–66.0)	0.048
Time with MAP ≤ 65 mmHg (min)	15.0 (13.0–23.0)	7.5 (10.0, 15.0)	0.012
Time with MAP ≥ 90 mmHg (min)	5.0 (12.0–20.0)	10.0 (13.0–25.0)	0.275
Time with HR ≤50 mmHg (min)	0.0 (0.1–1.1)	0.0 (0.0-0.9)	0.730
Time with HR ≥90 bpm (min)	0.0 (12.0–38.0)	0.0 (10.0–30.0)	0.938
*Postoperative outcomes*			
QoR-40 Questionnaire Score	164.0 (158.0-166.0)	160.5 (152.0-163.0)	0.267
Length of hospital stay (days)	2.0 (2.0, 2.0)	2.0 (2.0, 2.0)	0.222

## Discussion

The main finding of our study is that the administration of pregabalin at a dose of 150 mg prior to LSG demonstrates acceptable tolerability, yet did not have a significant impact on total opioid consumption or on secondary outcomes in a clinically important manner. The observed improvement in pain score at the 6th hour is of limited significance, as the difference did not reach the recognized MCID of 1 point on the NRS scale and was observed only at one predefined time point^12^.

The literature evaluating the efficiency of pre-emptive pregabalin administration in LSG and its impact on opioid requirements, pain scores, and side effects is scarce, and our study is among the few that assesses pregabalin as an element of a multimodal opioid-sparing strategy. Cabrera Shulmeyer et al.^14^ demonstrated clinically significant superiority of pregabalin 150 mg over placebo, with a 50% reduction in morphine requirements, lower pain scores, and decreased antiemetic use. Nonetheless, these findings did not translate into an overall impact on recovery^14^. Such a pronounced effect and the disparity between our studies may be partially attributed to the absence of other multimodal analgesic techniques and to the lack of PCA in postoperative care in their study protocol.

In line with the aforementioned study, Salama et al.^15^ demonstrated an even greater 68% reduction in total opioid dosage when administering pregabalin at 75 mg in conjunction with a dexmedetomidine infusion at 0.4 µg/kg/h. Nevertheless, within the scope of this study, it is not feasible to differentiate between the effects of the two medications, as dexmedetomidine has also been proven to possess opioid-sparing properties^16^.

Two other RCTs assessed pre-emptive administration of gabapentin, an analogous drug with a similar mechanism of action, in patients undergoing LSG^4,7^. Consistent with our findings, an RCT conducted by Rupniewska-Ładyko demonstrated promising opioid-sparing effects of a similar GABA receptor agonist, gabapentin, administered as a single oral dose of 1200 mg^4^, resulting in approximately a 20% reduction in oxycodone PCA doses and a delay in the time to first analgesic requirement. The authors also reported good tolerability; however, they did not examine typical side effects of gabapentinoids, such as oversedation and delayed recovery. Even more encouraging outcomes were observed by Khan et al.^7^, who used the same dose of gabapentin as in the aforementioned study and reported a 45% reduction in PCA morphine consumption within the study. Their RCT also assessed common adverse effects, which were comparable across groups. Notably, in the aforementioned studies, patients did not receive dexamethasone, which is well-documented to have an opioid-sparing effect in bariatric surgery^17^, potentially accounting for the more significant difference observed between the gabapentinoid and control groups. Furthermore, the 1200 mg dose of gabapentin utilized in the RCTs, as mentioned earlier, seems to be higher than an equivalent pregabalin dose in our study^18^; however, due to differences in pharmacokinetics, direct comparisons are challenging and may vary depending on specific aspects^19^.

In our study, pregabalin demonstrated a limited effect on circulatory parameters, with no significant differences observed in total ephedrine consumption, volume of administered fluids, or incidence of bradycardia; however, there was an increase in hypotension duration and lowest mean arterial pressure. This is of potential concern as intraoperative hypotension may be associated with increased risk of perioperative myocardial infarction or kidney failure^20^. This effect of pregabalin administration may be attributed to the synergistic interaction with remifentanil^21^. Regarding potential bradycardia and increased vasopressor requirements, pregabalin, as a co-analgesic agent, may be more beneficial for circulatory stability than agents such as lidocaine, dexmedetomidine, or the OFA technique^22–24^. However, exploration of this aspect would require a direct comparison between the co-analgesics.

In many centers, laparoscopic bariatric surgery is performed in accordance with ERAS guidelines^3^, focusing on rapid recovery and shortening the length of hospital stay, with some surgeries performed in selected groups as outpatient procedures^25^. In such a strategy, minimizing opioid use while providing adequate analgesia is essential, and our research aimed to assess pregabalin as an element of such a strategy. Currently, although potentially beneficial, gabapentinoids are not recommended as opioid-sparing co-analgesics in bariatric surgery due to the risk of associated side effects^6^. Our study demonstrated that preoperative pregabalin 150 mg does not result in clinically significant differences in side-effect prevalence, such as visual disturbances, oversedation, episodes of desaturation, or length of hospital stay. Nonetheless, although pregabalin was well tolerated, our research provides evidence that its administration as a co-analgesic does not confer clinically significant benefits to patients. Therefore, it cannot be recommended at a dose of 150 mg based on our findings in modern bariatric perioperative care.

The primary limitation of our study is its small sample size, which may have limited our ability to identify modest yet clinically significant opioid-sparing benefits. The efficacy of pregabalin was less than the assumed 30% opioid consumption reduction, and the study was underpowered to detect differences in specific clinical parameters. Furthermore, as the research was conducted at a single center, the generalizability of the findings may be restricted. Another limitation is that our RCT assessed only a single 150 mg dose. In contrast, a higher or repeated dose may be more effective, although it could be associated with an increased incidence of dose-related side effects. Furthermore, although the BMI distribution demonstrated no significant difference following statistical adjustment between the control and study groups, we cannot entirely exclude the possibility that the imbalance may still be a confounding factor, thereby introducing bias.

Consequently, further research in this area should focus on larger-scale multi-center RCTs and dose-finding studies. Additionally, it should aim to provide evidence for comparing pregabalin with other co-analgesics, as well as opioid-sparing and opioid-free techniques.

## Conclusions

The study demonstrated that the administration of oral pre-emptive pregabalin, 150 mg, in patients with obesity undergoing LSG as an element of an opioid-sparing strategy was well tolerated, yet did not have a significant impact on total opioid consumption or on secondary outcomes in a clinically important manner. The differences observed between the studied groups at specific time points do not support the recommendation of low-dose pregabalin as a routine medication for obese patients undergoing bariatric surgery.

## Data Availability

The datasets generated during and/or analyzed during the current study are available from the corresponding author upon reasonable request.
